# In Situ Phytoremediation of Mine Tailings with High Concentrations of Cadmium and Lead Using *Dodonaea viscosa* (Sapindaceae)

**DOI:** 10.3390/plants14010069

**Published:** 2024-12-29

**Authors:** Luis Fernando Acosta-Núñez, Patricia Mussali-Galante, María Luisa Castrejón-Godínez, Alexis Rodríguez-Solís, Joel Daniel Castañeda-Espinoza, Efraín Tovar-Sánchez

**Affiliations:** 1 Maestría en Manejo de Recursos Naturales, Universidad Autónoma del Estado de Morelos, Av. Universidad No. 1001, Col. Chamilpa, Cuernavaca 62209, Morelos, Mexico; acostaluis71095@gmail.com; 2 Laboratorio de Investigaciones Ambientales, Centro de Investigación en Biotecnología, Universidad Autónoma del Estado de Morelos, Av. Universidad No. 1001, Col. Chamilpa, Cuernavaca 62209, Morelos, Mexico; alexis.rodriguez@uaem.mx; 3 Facultad de Ciencias Biológicas, Universidad Autónoma del Estado de Morelos, Av. Universidad No. 1001, Col. Chamilpa, Cuernavaca 62209, Morelos, Mexico; mlcastrejon@uaem.mx; 4 Departamento de Interacción Planta-Insecto, Laboratorio de Entomología, Centro de Desarrollo de Productos Bióticos, Instituto Politécnico Nacional, Carretera Yautepec-Jojutla Km 6, Calle Ceprobi No. 8, Col. San Isidro, Yautepec 62731, Morelos, Mexico; daniel_rojo8@hotmail.com; 5 Centro de Investigación en Biodiversidad y Conservación, Universidad Autónoma del Estado de Morelos, Av. Universidad No. 1001, Col. Chamilpa, Cuernavaca 62209, Morelos, Mexico

**Keywords:** biochar, mine tailing, phytostabilization, genetic damage, hyperaccumulator

## Abstract

The waste generated during metal mining activities contains mixtures of heavy metals (HM) that are not biodegradable and can accumulate in the surrounding biota, increasing risk to human and environmental health. Plant species with the capacity to grow and develop on mine tailings can be used as a model system in phytoremediation studies. *Dodonaea viscosa* (L.) Jacq. is a shrub with wide geographical distribution and the ability to establish itself in mine tailings. The Sierra de Huautla Biosphere Reserve in Mexico contains a metallurgic district where mining activities have generated 780 million kg of waste with large concentrations of toxic heavy metals, mainly cadmium and lead. The present study evaluated the phytoremediation potential of *D. viscosa* in in situ conditions on soils contaminated with HMs (exposed) and reference sites (non-exposed) for one year. Also, the effects of cadmium (Cd) and lead (Pb) exposure in *D. viscosa* were analyzed via DNA damage (comet assay) morphological and physiological characters in exposed *vs* non-exposed individuals. The concentration of Cd and Pb was measured through atomic absorption spectrophotometry in the roots and leaves of plants. In total, 120 *D. viscosa* individuals were established, 60 growing in exposed and 60 in non-exposed soils. Exposed individuals of *D. viscosa* hyperaccumulated Cd and Pb in roots and leaves. At the end of the experiment, eight out of twelve characters under evaluation decreased significantly in HM-exposed plants in relation to individuals growing in non-exposed soils, except for stomatal index, stomatal coverage, and fresh leaf biomass. The micro-morphological and physiological traits of *D. viscosa* were not influenced by Cd and Pb bioaccumulation. In contrast, the bioaccumulation of Cd and Pb significantly influenced the macro-morphological characters and genetic damage; this last biomarker was 3.2 times higher in plants growing in exposed sites. The bioconcentration factor (BCF) of Cd and Pb in root and leaf tissue increased significantly over time. The mean BCF in root and leaf tissue was higher for Pb (877.58 and 798.77) than for Cd (50.86 and 23.02). After 12 months of exposure, *D. viscosa* individuals growing on mine tailing substrate showed that the total HM phytoextraction capacity was 7.56 kg∙ha^−1^ for Pb and 0.307 kg∙ha^−1^ for Cd. *D. viscosa* shows potential for phytoremediation of soils contaminated with Cd and Pb, given its capacity for establishing and developing naturally in contaminated soils with HM. Along with its bioaccumulation, biomass production, abundance, and high levels of bioconcentration factors, but without affecting plant development and not registering associated herbivores, it may incorporate HM into the trophic chain.

## 1. Introduction

Mine tailings resulting from mining activities are a major source of solid toxic waste. Globally, more than 800 million m^3^ of mine tailings are produced each year [1], which are of environmental concern due to potential hazards of soil, surface, or groundwater pollution, posing a threat to environmental and human health [2]. Mine tailings are a major source of toxic HMs since they are not metabolically assimilated, thus bioaccumulating in different organisms, resulting in detrimental effects across all levels of biological organization [3]. In terrestrial environments, HM bioaccumulation in plants is the first step towards their incorporation into the trophic webs [4], which depends on (a) soil physicochemical conditions, like pH, particle size (<0.02 mm), organic matter content, cationic exchange capacity and type of soil microorganisms, among others [5], and (b) two general plant strategies to cope with HM exposure; HM evasion capacity, trough impermeability and/or metal excretion and HM accumulation and tolerance [6]. Once bioaccumulated, HM can exert several toxic effects on plants; three general mechanisms have been identified: (1) production of reactive oxygen species, which may damage important cellular components and macromolecules; (2) inhibition of key functional groups in proteins and other target molecules and (3) displacement of essential metal ions from biomolecules [7,8]. These effects can result in micro and macromorphological alterations, changes in total biomass and in biochemical disruption [9,10]. Moreover, HM bioaccumulation in plants causes cytotoxic and genotoxic damage, which in turn results in cellular, physiological, and morphological alterations [11] and growth inhibition [12].

In particular, lead and cadmium are two of the most toxic metals found in soils polluted by mining activities, and they are commonly found in abandoned and active mine sites. These HMs are non-trace elements because they do not have an essential role in organisms, owing to their heavy toxicity at low concentrations [13]. To cope with HM stress, some plant species have developed the capacity to absorb and bioaccumulate high non-trace HM concentrations in their tissues.

In the last decades, these plant strategies have been employed to remove, detoxify, or immobilize HM from soils using different species, especially hyperaccumulating plants, a method known as phytoremediation [6,14]. Furthermore, the selected species need to have a high biomass production, fast growth rates and high capacity to grow and survive on HM-polluted soils [15]. Phytoremediation has been a reliable tool to remediate heavily polluted soils because it is economically viable, improves soil characteristics, can be used in great soil extensions and has less damaging environmental effects compared to other remediation techniques [6]. Additionally, the results of in situ phytoremediation strategies are more easily extrapolated to other polluted soils from different sites.

Recently, *D. viscosa* has been recognized to bioaccumulate different HMs, like Fe, Cd, Cr, Ni, Cu, Mn, Pb, and Zn [12,16,17,18]. It has a wide geographical distribution, and it is established in perturbed sites [19]. In some vegetation types, such as xeric scrub, *D. viscosa* is one of the species that exhibits the highest biomass production all year long [20] without being affected by HM bioaccumulation [12]. In addition, *D. viscosa* is recognized as a soil-forming species [21]. Hence, the present study evaluated the capacity of *D. viscosa* to bioaccumulate and translocate Cd and Pb in situ at an exposed (mine tailings) and a control site. Also, the effects of Cd and Pb exposure on *D. viscosa* were analyzed through (a) DNA damage, measured as single strand DNA breaks; (b) by physiological processes; total content of chlorophyll a and b and (c) by analyzing the micro and macro-morphological alterations in exposed vs non-exposed individuals.

## 2. Results

### 2.1. Morphological, Physiological and Genetic Damage in D. viscosa Plants Growing on Mine Tailing

#### 2.1.1. Macro-Morphological Characters

**Figure 1:**
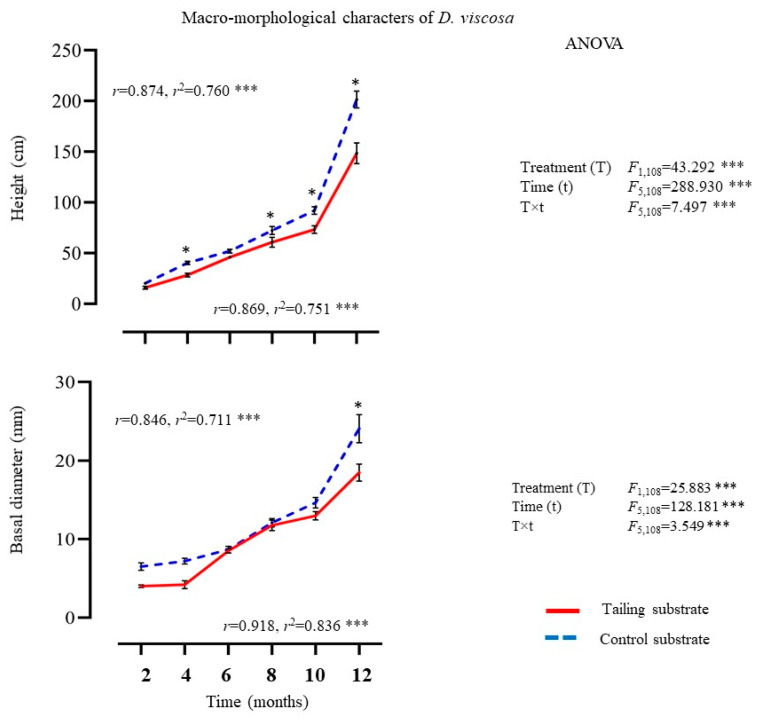

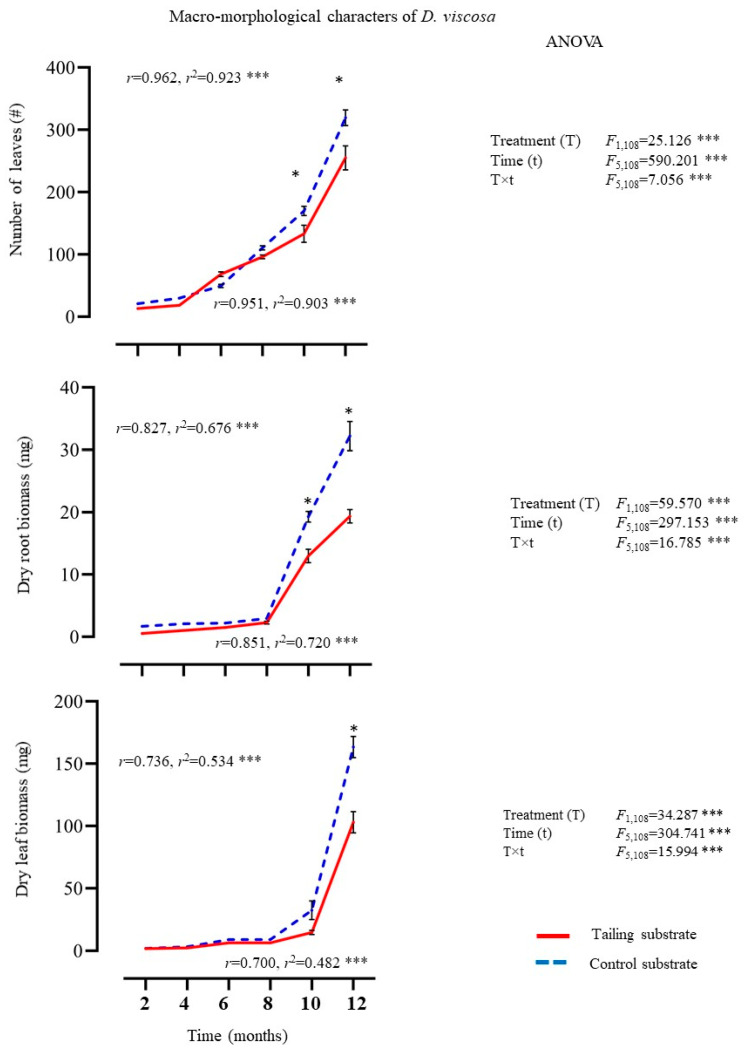

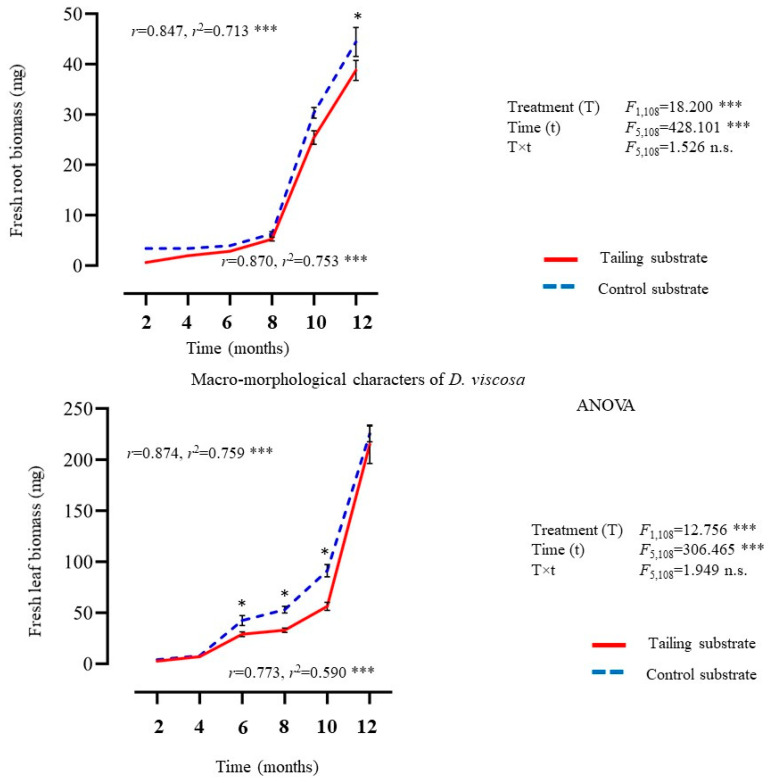
Average ± standard deviation of macro-morphological characters from individuals of *D. viscosa* growing under in situ conditions on mine tailing and control sites. Two-way ANOVA to evaluate the effect of time (12 months) and treatment (mine tailing and control substrate) on macro-morphological characters. Regressions analysis between exposure time to the treatment and macro-morphological characters. The single asterisk denotes significant differences between treatments by exposure time with *p* < 0.05 (Tukey). ANOVA test: *** = *p* < 0.001, n.s. = not significant differences.

The two-way analysis of variance documented a significant effect of the treatment, time, and their interaction (treatment × time) on macro-morphological characters evaluated in individuals of *D*. *viscosa* (Figure 1). In general, the plants of *D. viscosa* growing on mine tailings showed no significant differences in macro-morphological characters with respect to the individuals of the control substrate, at least not until the end of the experiment, when the plants growing in the control substrate showed higher values. However, higher plant height was registered in the individuals growing on the control substrate. Moreover, fresh leaf biomass showed no significant differences between treatments at the beginning and end of the experiment. However, a positive and significant relationship between exposure time and macro-morphological characters was detected for both treatments (Figure 1).

#### 2.1.2. Micro-Morphological Characters

**Figure 2:**
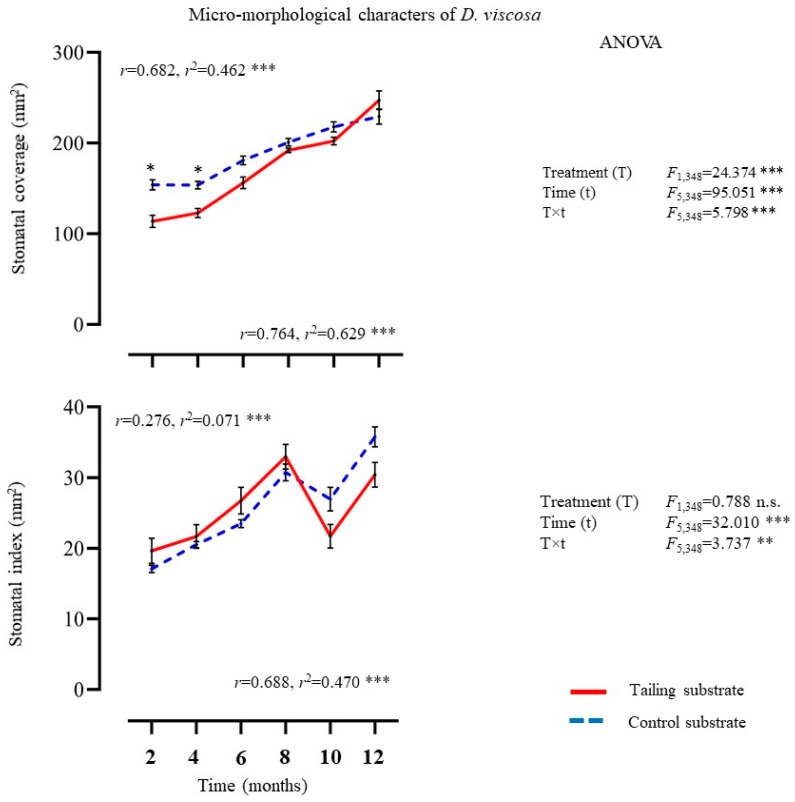
Average ± standard deviation of micro-morphological characters from individuals of *D. viscosa* growing under in situ conditions on mine tailing and control sites. Two-way ANOVA to evaluate the effect of time (12 months) and treatment (mine tailing and control substrate) on micro-morphological characters. Regressions analysis between exposure time to the treatment and micro-morphological characters. The single asterisk denotes significant differences between treatments by exposure time with *p* < 0.05 (Tukey). ANOVA test: *** = *p* < 0.001, ** = *p* < 0.01, n.s. = not significant differences.

The micro-morphological characters in *D. viscosa* plants varied significantly between treatments, among exposure times, and their interaction (treatment × time) (Figure 2). In general, the Tukey test showed no significant differences between treatments at any time, except at 2 and 4 months for stomatal coverage. However, a positive and significant relationship between exposure time and micro-morphological characters was detected for both treatments (Figure 2).

#### 2.1.3. Physiological Characters

**Figure 3:**
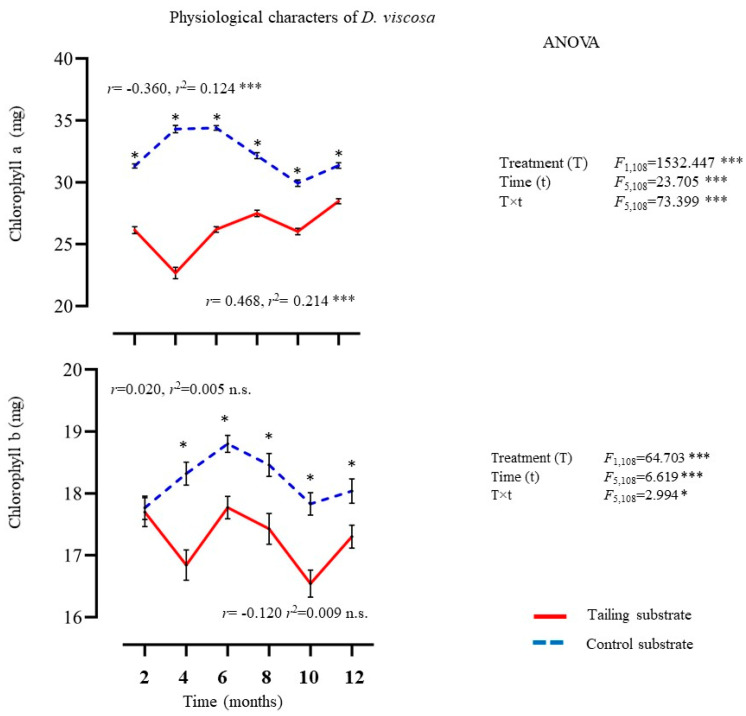
Average ± standard deviation of physiological characters from individuals of *D. viscosa* growing under in situ conditions on mine tailing and control sites. Two-way ANOVA to evaluate the effect of time (12 months) and treatment (mine tailing and control substrate) on physiological characters. Regressions analysis between exposure time to the treatment and physiological characters. The asterisks denote significant differences between treatments by exposure time with *p* < 0.05 (Tukey). ANOVA test: *** = *p* < 0.001, * = *p* < 0.05, n.s. = not significant differences.

Chlorophyll *a* and *b* in leaves of *D. viscosa* differed significantly between treatments, time, and their interaction (treatment × time) (Figure 3). Chlorophyll levels were higher in individuals of *D. viscosa* growing on the control substrate. Regression analysis detected a positive and significant relationship between exposure time and chlorophyll *a* in *D. viscosa* individuals growing on mine tailing substrate. In contrast, individuals growing on the control substrate recorded an inverse pattern. No significant correlation at *p* < 0.05 was detected for chlorophyll *b* in plants of *D. viscosa* growing on both treatments (Figure 3).

#### 2.1.4. Genetic Damage (DNA Single Strand Breaks)

**Figure 4:**
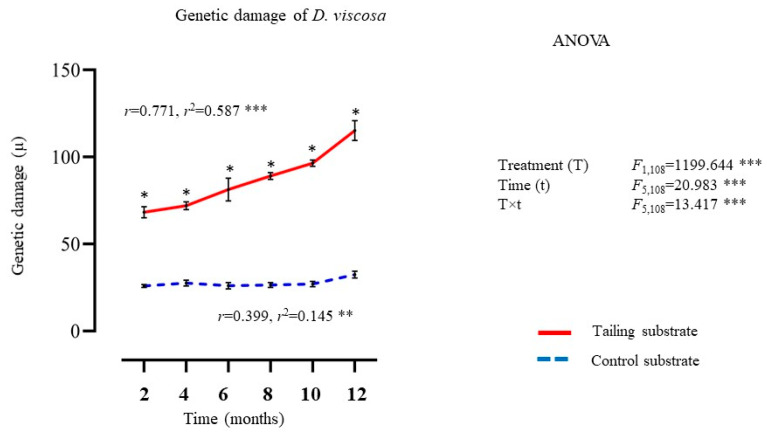
Average ± standard deviation of genetic damage from individuals of *D. viscosa* growing under in situ conditions on mine tailing and control sites. Two-way ANOVA to evaluate the effect of time (12 months) and treatment (mine tailing and control substrate) on genetic damage. Regressions analysis between exposure time and genetic damage. The single asterisk denotes significant differences between treatments by exposure time with *p* < 0.05 (Tukey). ANOVA test: ** = *p* < 0.01, *** = *p* < 0.001.

In general, a significant effect of treatment, time, and their interaction (treatment × time) on DNA damage levels was detected (Figure 4). DNA damage levels were higher in the plants of *D. viscosa* growing on mine tailings compared to individuals growing on the control substrate. Regression analysis showed a positive and significant relationship between exposure time and DNA damage levels in individuals growing on both treatments (Figure 4).

### 2.2. Relationship Between Heavy Metal Bioaccumulation and Micro-, Macro-Morphological, Physiological, and Genetic Damage in Plants of D. viscosa

In general, Pb and Cd bioaccumulation in *D. viscosa* individuals modified their macro-morphological characters: Pb bioaccumulation in dry and fresh leaf biomass and Cd bioaccumulation in leaves. Specifically, Pb bioaccumulation had a significant and negative effect on the height, basal diameter, number of leaves, and dry and fresh root biomass of *D. viscosa*, which is explained by 7.3%, 31.9%, 40.7%, 14.5%, and 32.1% of the variation, respectively (Table 1). Cd bioaccumulation had a significant negative effect on the height, basal diameter, dry and fresh root biomass, and dry and fresh leaf biomass of *D. viscosa*, which is explained by 72.2%, 9.6%, 79.2%, 67.2%, 54.7% and 55.9% of the variation, respectively (Table 1). In contrast, heavy metal bioaccumulation in *D. viscosa* plants did not influence their micro-morphological and physiological characteristics (Table 1). Finally, Cd and Pb bioaccumulation had a significant and positive effect on the induction of DNA single breaks in *D. viscosa*, as shown by 18.1% and 9.3% of the variation, respectively (Table 1).

### 2.3. Heavy Metal Enrichment in the Roots and Leaves and Translocation Factor of D. viscosa Plants Growing on Mine Tailings

In general, regression analysis documented a positive and significant relationship between mine tailing exposure time and Pb, Cd, BCF_root_, and BCF_leaf_ values. In contrast, no correlation was documented between mine tailing exposure time and TF for Pb and Cd (Table 2). The mean bioconcentration factor values (BCF) in root and leaf tissue were higher for Pb (877.58 and 798.77) than for Cd (50.86 and 23.02).

### 2.4. Total Heavy Metal Phyto-Extraction in D. viscosa Individuals

After 12 months of exposure, *D. viscosa* individuals growing on mine tailing substrate showed that the total heavy metal phytoextraction (mean ± s.d.) capacity was 7.56 ± 2.09 kg∙ha^−1^ for Pb and 0.307 ± 0.081 kg∙ha^−1^ for Cd. According to the model of plant species behavior in soils with heavy metals, *D. viscosa* could be considered a hyperaccumulator species for Pb and Cd (Table 3).

## 3. Discussion

Although some studies have documented the levels of enrichment, translocation, and effects of HM exposure in *D. viscosa* individuals, these have been carried out under controlled experimental conditions, such as in greenhouses. Therefore, this study contributes to documenting the effects of HM exposure based on a multi-biomarker approach, with the aim of evaluating the potential of *D. viscosa* as a species capable of phytoremediation of polluted soils with Cd and Pb under in situ conditions.

### 3.1. Lead and Cadmium Bioaccumulation and Translocation Factors in Individuals of D. viscosa

This study noted that *D. viscosa* individuals growing on mine tailings bioaccumulated Cd and Pb in roots and leaves, showing a bioaccumulation pattern for both tissues Pb > Cd. In particular, high concentrations of Pb were detected in both tissues, especially in the root, with an exponential increase over time. These results are consistent with those reported by Rojas-Loria et al. [22] and Castañeda-Espinoza et al. [12], who document the ability of *D. viscosa* to accumulate Cd and Pb, mainly in the root. This bioaccumulation pattern is like other plant species that grow on polluted soils with HMs, such as *Acacia robeorum* Maslin [23] *Prosopis laevigata* Humb. et Bonpl. ex Willd [9,24], *Vachelia campechiana* D (Mill.) Seigler and Ebinger [10], *Gliricidia sepium* (Jacq.) Kunth ex Walp. [15], and *Crotalaria pumila* Ortis [25], all belonging to the Fabaceae family.

In general, the values obtained for the translocation factor in *D. viscosa* individuals for some essential (Fe and Zn) and non-essential (As, Pb) elements have been documented, which showed for Pb TF < 1 [12,22]. The above is consistent with this study since Cd and Pb registered TF < 1 regardless of the sampling date. This phenomenon suggests that *D. viscosa* has the ability to retain Pb and Cd in the roots under in situ conditions, limiting their mobilization towards the aerial part and resulting in low TF values. In this sense, Liu et al. [26], Castañeda-Espinoza et al. [12], and Santoyo-Martínez et al. [27] documented a significant Pb accumulation in the roots of *Brassica juncea*, *D. viscosa*, and *Crotalaria. pumila* respectively, compared to the tissues of the aerial part. Moreover, Rosas-Ramírez et al. [25] showed a significant accumulation of Pb and Cd in the roots of *C. pumila* compared to the leaf tissue. This strategy of Pb and Cd exclusion in the roots is characteristic of tolerant species to heavy metals, thus reducing the possible damage these metals could cause in plants [28].

### 3.2. Micro, Macro-Morphological, Physiological Traits, and Genetic Damage Changes in D. viscosa Plants Growing on Exposed and Non-Exposed Heavy Metal Substrate over Time, Under In Situ Conditions

#### 3.2.1. Micro-Morphological Characters

In this study, the stomatal index and coverage did not differ significantly between plants of *D. viscosa* growing on exposed sites in comparison with individuals growing on non-exposed sites under in situ conditions, except for stomatal coverage at two and four exposure months, which was significantly higher in non-exposed plants. Moreover, multiple regression analysis did not detect an influence of Pb and Cd bioaccumulation on these micro-morphological characters (stomatal index and stomatal coverage). These results are supported by the study of Muro-González et al. [9] for *P. laevigata* growing on mine tailing substrate under greenhouse conditions. In their study, plants exposed to HMs did not show significant differences in stomatal coverage in comparison to individuals growing on the reference substrate. In general, this may have occurred because plants exposed to Cd and Pb may present anatomical and physiological variations as adaptive responses to influence pollutant disturbance conditions [29].

#### 3.2.2. Macro-Morphological Characters

In general, Pb and Cd bioaccumulation in *D. viscosa* plants growing on exposed sites under in situ conditions showed a significant reduction of macro-morphological characters in comparison with the individuals growing on the non-exposed substrate. In general, *D. viscosa* plants showed a reduction in height, basal diameter, number of leaves, and biomass. Bioaccumulation of Pb and Cd can cause different macro-morphological alterations in plants. The more frequent effects reported include (1) growth inhibition [10,24], reduction in foliar biomass [9,12,15,24], and alterations in leaf shape [30]. The same patterns have been documented in other plant species, such as *P. laevigata* [9], *V. campechiana* [10], *G. sepium* (Fabaceae) [15], *C. pumila* [25,27], *Arundo donax* (Poaceae) [31,32] and *D. viscosa* [12]. In general, the plants were all growing on soils contaminated with a mixture of HMs, including Pb and Cd. Pb and Cd bioaccumulation could affect metabolic processes and are potentially toxic [33]. This phytotoxicity could result in growth alterations along with weak plant growth and yield depression [34]. These effects can be related to metabolism disorders such as the reduction of the meristematic zone [35].

#### 3.2.3. Physiological Parameters: Chlorophyll Content

In general, *D. viscosa* plants growing under in situ conditions registered a significant effect of the treatment (exposed and non-exposed to HM) on chlorophyll content (*a* and *b*). The present results are supported by what is documented in *Sambitalia procumbens* [36], *P. laevigata* [9], *C. pumila* [25], where there was a reduction of chlorophyll content in plants exposed to Cd and/or Pb contained in mine tailing substrate. Similarly, other studies showed that Pb bioaccumulation in plants registered a reduction in chlorophyll concentration [37] and a reduction in photosynthetic pigments [38].

Bioaccumulation of HMs in plants, such as Pb and Cd, could modify the photosynthetic functions that repress chlorophyll biosynthesis [39], decreasing the total proportion of chlorophyll *a* and *b*, which may result in a reduction in the photosynthetic rate [40]. For example, it has been documented that Pb can substitute essential elements such as Mg in plants. As a consequence, it inhibits the synthesis of chlorophyll *a* and *b*, generating a decrease in photosynthetic activity and electron transport [41]. In addition, in electron transport systems, Pb can alter the donor and receiving sites of the photosystem II. Furthermore, Pb can replace the Mg ion of the chlorophyll molecule, which makes the capture of photons nonviable, promoting a reduction in photosynthetic activity [42].

On the other side, Cd can negatively influence photosynthesis even at low concentrations. Cadmium reduces chlorophyll synthesis, which can generate chlorosis in the leaves, reducing the carotenoid content and altering the transpiration rate [43]. Also, Cd bioaccumulation produces instability in chloroplast metabolism, inhibiting chlorophyll synthesis and decreasing the activity of enzymes implicated in CO_2_ fixation [44]

In summary, the current results suggest that non-essential elements, such as Cd and Pb, which are toxic elements at low concentrations, negatively affect chlorophyll synthesis, which in turn affects photosynthesis in *D. viscosa* individuals.

#### 3.2.4. Genetic Damage

In this study, *D. viscosa* individuals growing on mine tailing registered 3.2 times more genetic damage than those non-exposed to HMs. Similar results have been documented in *Nicotiana tabacum* [45], *P. laevigata* [9,24], *D. viscosa* [12], and *C. pumila* [25]. It has been documented that non-essential metals, such as Cd and Pb, are high precursors of genetic damage when they are bioaccumulated in plants. HM can promote the induction of DNA breaks in different ways, both directly and indirectly; Pb and Cd may arrive at the cell nucleus and bind to the purine and pyrimidine bases, producing single- and double-stranded DNA breakage, leading to genetic damage [46].

It has also been widely documented that Pb and Cd induce oxidative stress, enhancing the production of Reactive Oxygen Species, increasing the level of free radicals in the cells, and negatively affecting DNA [47]. In particular, Pb and Cd are high precursors of genetic damage when they are bioaccumulated in plants. Lead indirectly leads to single-strand DNA breakage; this element could also cause oxidative stress, as well as replace Zn in the repair and replication enzymes with zinc fingers [40], generating genetic damage. Regarding Cd, it generates oxidative stress, which causes proteins and membrane modifications [48], as well as DNA base pair oxidations that influence single- and double-stranded DNA breakage [44]. Therefore, the bioaccumulation of Pb and Cd in individuals of *D. viscosa* suggests that these elements could be the main reason for the genetic damage observed.

#### 3.2.5. Bioconcentration Factor of Cd and Pb in Roots and Leaves and Translocation Factor in *D. viscosa* Individuals Growing Under In Situ Conditions

*D. viscosa* is a plant species with a wide geographic distribution in Mexico and is frequently associated with disturbed sites, such as HM-polluted soils [12,19]. In this study, *D. viscosa* plants established in mine tailing that bioaccumulate Pb and Cd in root and leaf tissues showed the following bioaccumulation pattern for both tissues: Pb > Cd. In general, the mean bioconcentration factor values (BCF) in root and leaf tissues of *D. viscosa* for Cd and Pb bioaccumulation was >1 in plants growing on the mine tailing substrate. The BCF in root and leaf tissues was higher for Pb (877.58 and 798.77) than for Cd (50.86 and 23.02). Also, regression analysis documented a positive and significant relationship between mine tailing exposure time and Pb, Cd, BCF_root_, and BCF_leaf_ values. In particular, these results evidenced that the individuals of *D. viscosa* growing under in situ conditions of HMs-contaminated soils showed higher bioaccumulation and BCF of Pb and Cd in root and leaf tissues. The above suggests that plants of *D. viscosa* with a BCF value > 1 are potentially useful for the phytoremediation of soils polluted with Cd and Pb and for the phytoextraction of HMs [49]. These levels are similar to the BCF in species considered accumulators that grow directly on contaminated soils, such as *V. campechiana* (BCF_root_ Pb = 15.1, BCF_leaf_ Pb = 17.4) [10]. *P. laevigata* (BCF_root_ Pb = 3.48, BCF_leaf_ Pb= 7.04) [9], *C. pumila* (BCF_root_ Pb = 147.34, BCF_leaf_ Pb = 49.52; BCF_root_ Cd = 14.64, BCF_leaf_ Cd = 5.44) [25], and *D. viscosa* (BCF_root_ Pb = 8.53, BCF_leaf_ Pb = 1.32; BCF_root_ Cd = 1.88, BCF_leaf_ Cd = 1.22) [12].

The current results showed that Pb bioaccumulation in both tissues (roots and leaves) of the *D. viscosa* individuals growing under in situ conditions increased over time, registering higher bioaccumulation levels in roots. Lead bioaccumulation in leaf tissue has been documented in different plant species, such as *D. viscosa* [12], *C. pumila* [25], *P. laevigata* [9], *V. campechiana* [10], *Zea mays* [30], *Acacia hafneri* (S. Watson) F.J.Herm (Fabaceae) [50], *Brickellia veronicifolia* (Kunth) A.Gray (Asteraceae) [51], *Buddleja scordioides* unth (Scrophulariaceae), and *Mimosa aculeaticarpa* Ortega (Fabaceae) [50], among others. Lead bioaccumulation affects the transport of essential elements, producing an accumulation in its concentration and translocation levels over time in leaves.

Cadmium bioaccumulation in both tissues (roots and leaves) of *D. viscosa* individuals showed that concentration increased significantly over time, showing the bioconcentration was higher in roots than in leaves. This result is supported by those documented in *C. pumila* [25], *Matisia cordata* Bonpl and *Theobroma cacao* (L.), *Malvaviscus* sp. Fabr. (Malbaceae) [52]. For this reason, it has been suggested that Cd bioaccumulates, particularly in the plant roots and is sequestered in the vacuole of the cells, and only a small part is carried to the aerial part [53].

In this study, the density of *D. viscosa* plants obtained in natural conditions in the field, equivalent to 10,800 plants per hectare, was considered for total HM phytoextraction determinations. After 12 months of exposure, *D. viscosa* individuals growing on the mine tailing substrate under in situ conditions showed a total HM phytoextraction value (mean ± s.d.) of 7.56 ± 2.09 kg∙ha^–1^ for Pb and 0.31 ± 0.08 kg∙ha^–1^ for Cd. Also, in phytostabilization studies, arrangements with closer distances between plants have been tested to produce higher dry-weight biomass and, subsequently, the highest values for the total phytoextraction of HM [54,55]. As a result, arrangements with a higher number of *D. viscosa* plants in the field can increase Cd and Pb phytoextraction and phytostabilization in phytoremediation field experiments.

## 4. Materials and Methods

### 4.1. Study Area: Huautla and Quilamula, Morelos

The present study was carried out in the mine tailings (treatment: mine tailing) located in Huautla, Morelos, Mexico (18°25′24″ N and 99°01′44″ W), a site within the Sierra de Huautla Biosphere Reserve (REBIOSH, acronym in Spanish). The mine tailings are located at 1004 m above sea level; the tailings substrate has the following physicochemical characteristics: 11.1% humidity, 0.56% organic matter (O.M.), pH of 7.1, electrical conductivity (EC) of 0.17 (dS cm^−1^), and particle size < 45 µm. In this site, approximately 780 million kg of mining wastes are found in this area, with significant concentrations of toxic metalloids (As) and heavy metals (Cd, Cr, Cu, Fe, Pb, Mn, and Zn), Pb (1017.5 mg∙kg^−1^) and Cd (19.0 mg∙kg^−1^) exceed the maximum permissible limits for soils according to the US Environmental Protection Agency (EPA), which represent a risk to the environmental and public health. The bioavailable concentrations of Cd and Pb in the mine tailings were 8.365 mg∙kg^−1^ and 6.972 mg∙kg^−1^, respectively.

**Figure 5:**
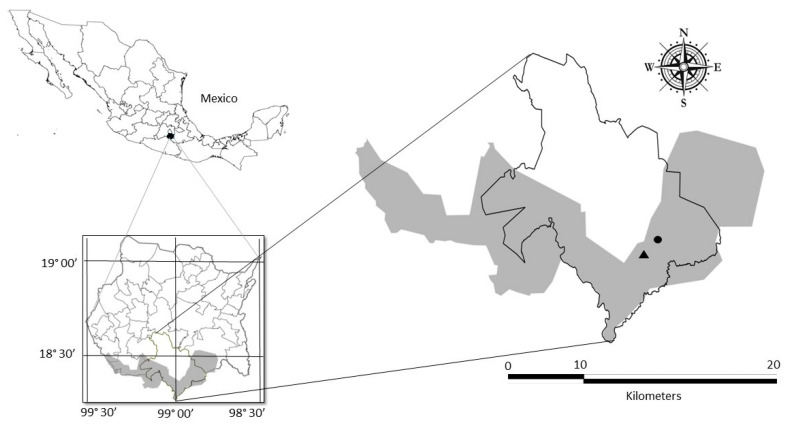
Study sites within the Sierra de Huautla Biosphere Reserve (gray shaded polygon), the black triangle indicates the location of the exposed site (mine tailings), and the black circle indicates the location of the control site (Quilamula town).

On the other hand, the control site (treatment: control) was located in Quilamula, Morelos, Mexico (18°30′4.1″ N–98°59′52.6″ W and 18°32′12.2″ N–99°02′05″ W), also located at the REBIOSH. The control site is located at an altitude of 983 m; the soil has the following physicochemical characteristics: 21.4% humidity, 6.44% O.M., pH of 6.7, EC is 0.25 (dS cm^−1^). It has the same geographic and ecological characteristics as the mine tailing sites but without records of mining activity. The reference locations are approximately 7.5 linear kilometers from the exposed sites (Figure 5).

### 4.2. Study Species

*Dodonaea viscosa* is a shrub species native to Australia, currently distributed among the tropics and subtropics regions, especially in countries from Africa, India, and Mexico, as well as in the states of Florida and Arizona in the United States. According to Villaseñor and Espinosa [56], *D. viscosa* has a wide geographic distribution in Mexico; it is found at altitudes ranging from 0 to 2600 m [57]. *D*. *viscosa* individuals are large shrubs or small trees up to 3 m tall. It is an evergreen perennial species that bloom from September until December and fruits from November until April. Their seeds are black, subglobose, compressed, and 3 mm in diameter [57]. *D. viscosa* is a plant species that establishes, grows, and reproduces naturally in the mine tailings of Huautla, Morelos.

### 4.3. Seed Collection, Germination and Seedlings of D. viscosa

*D. viscosa* seeds were collected from plants established in the control site (Quilamula town). In total, twenty individuals were randomly sampled, and 20% of their seeds were collected according to the recommendations of Gold et al. [58]. The seeds were transported to the laboratory, and subsequently, they were cleaned and selected, removing the seeds parasitized by insects. For germination, the collected seeds were immersed in water at 75 °C for three minutes, afterward placed in water at room temperature (25 °C ± 3 °C) for 12 h [59]. A total of 180 seedlings (90 individuals per treatment: mine tailings and control) were transplanted into individual nursery polyethylene bags to reach adequate size for in situ experiments.

### 4.4. Experimental Plots

The in situ phytoremediation experiments were conducted in experimental plots with 90 plants per treatment (mine tailings and control site). The plants were transplanted at the control and mine tailings sites through a 200 m transect; *D. viscosa* plants of 5 cm average total height were planted in 20 cm depth holes, with a separation of 2 m between individuals. Each plant was irrigated twice weekly with tap water during the first six months. Ten plants were randomly sampled in each experimental treatment every two months until completed a year. Subsequently, macro- and micro-morphological characters, chlorophylls (*a* and *b*), and genetic damage were determined in each sampled plant (Table 4). Finally, the Cd and Pb concentrations in mine tailings of the exposed site and soil from the control site and in plant tissues (roots and leaves) were determined through atomic absorption.

### 4.5. Macro- and Micro-Morphological Characters Determinations

After sampling, the ten plants corresponding to each treatment were evaluated in seven macro-morphological characters, such as the total individual height, number of leaves, basal diameter, fresh and dry root biomass, and fresh and dry leaf biomass. To determine dry biomass, both tissues were cleaned with running tap water and then rinsed three times with distilled water to eliminate residual substrate. This was followed by oven drying at 60 °C until reaching a constant weight. Concerning micro-morphological features, the stomatal index and stomatal coverage were assessed. Fresh and clean leaf tissues were utilized to create foliar epidermal impressions using the cyanoacrylate glue method. Three impressions of the leaf’s abaxial side were made for each plant based on their respective treatment. The foliar epidermal impressions in microscope slides were examined at 40X through bright field illumination (BFI) and differential interference contrast (CDI) optical microscopy (Leica, Wetzlar, Germany); three photomicrographs were captured for each microscope slide to tally the quantity of stomatic and epidermal cells.

The Stomatic index was calculated according to Salisbury and Parke [60] (Equation (1)); the stomata and epidermal cells seen in the visual field were tallied, and only those stomas with two guard cells intact were taken into account; the number of epidermal cells was counted in at least 60% of the visual field, following the guidelines set by Paniagua-Ibáñez et al. [61].

Stomatic index = N.E./N.E. + N.C.E. × 100(1)

where:

N.E. = Number of stomatic cells (guard cells) per leaf unit
N.C.E. = Number epidermal cells per leaf unit

The Stomatic coverage was determined using Equation (2). The measurements of stoma length (larger diameter) and width (perpendicular diameter) in micrometers (μm) for six stomata per plant were taken. The stomatic coverage was reported in each sampling time, and treatment was reported as the average of the individual stoma coverage of the six stomata evaluated in μm^2^.

Stoma coverage = [(Q_1_ + Q_2_)/4]^2^ × π (2)

where:

Q_1_ = larger diameter (μm)
Q_2_ = perpendicular diameter (μm)

### 4.6. Determination of Chlorophyll Content

The chlorophyll index (chlorophyll *a* and *b*) (mg∙m^−2^) was measured in the 180 individuals (90 per treatment) for each exposure time (2, 4, 2, 8, 10, and 12 months), with a chlorophyll measuring device (ClorofiLOG, model CFL 1030, Falker, Porto Allegre, Brazil). All readings were taken at the center of the leaf blade. The chlorophyll measurements were always taken between 9:00 and 10:00 a.m. to avoid potential sunlight effects. Measured values are expressed in dimensionless units like the Falker Chlorophyll Index (FCI).

### 4.7. Genetic Damage: Alkaline Gel Electrophoresis (Comet Assay)

Leaves from 10 individuals of *D. viscosa* were chosen for each treatment (n = 60), showing no visible physical harm. The samples were rinsed with distilled water to eliminate dirt particles. After that, leaves were cut on a glass Petri dish with 200 µL of cold phosphate buffer saline (PBS, pH 7.4) under dim light conditions. Microscope slides were submerged in a 1% RMP (regular melting point) agarose solution in water at 50 °C. Once solidified, 50 µL of suspension with the isolated nuclei was mixed with 50 µL of a 1% LMP (low melting point) agarose solution (40 °C), prepared on PBS. The ultimate blend was placed on slides that had been prepared earlier. Then, a coverslip was positioned over the mixture and stored on ice. Once solidified, 80 µL of 0.5% LMP-agarose (37 °C) was added as a third layer, covered with the coverslip, and kept on ice for 5 min. After the coverslip was taken off, the slides were plunged into a cold lysis solution (100 mM Na_2_EDTA, 2.5 M NaCl, 10 mM fresh Tris, (pH 10), 1% Triton X100, and 10% dimethyl sulfoxide) and incubated at 4 °C for 24 h. Subsequently, the slides were positioned in a horizontal gel electrophoresis tank containing cold electrophoresis buffer (1 mM Na_2_EDTA and 300 mM NaOH, pH 13) for 15 min to let the DNA unwind. The electrophoresis process was performed at 300 mA and 25 V for 20 min at 4 °C without light. Next, the slides were neutralized (0.4 M Tris, pH 7.5) three times for 5 min and dehydrated with ethanol (100%). Ultimately, the slides were treated with 50 µL of ethidium bromide (20 µg mL^−1^) and visualized at 400X under a fluorescent microscope (Carl Zeiss, Jena, Germany), 515–560 excitation filter and a barrier filter of 590 nm. A computerized Image Analysis System (Comet Assay IV, Perceptive Instruments, Staffordshire, UK) was used to analyze fifty randomly selected nuclei from each slide (totaling 100 nuclei per sample). The parameters used to evaluate genetic damage levels were tail moment and tail length.

### 4.8. Cadmium and Lead Concentration in Root and Leaves Tissues of D. viscosa Individuals

The root and leaf tissues of *D. viscosa* plants were cleaned using running tap water and then rinsed with distilled water three times to eliminate any attached substrate residues. Clean tissues were dried in an oven until they reached a constant weight (60 °C, 72 h). Three samples of 0.25 g dry tissues from each structure were ground and placed in teflon containers for acid digestion using a Microwave Accelerated Reactions System (CEM^®^ MARS-5, Sineo, Shanghai, China); for acid digestion, a mixture of HNO_3_ (70%) and HCl (30%) was added to tissue samples. Samples were dissolved in distilled water and filtered to reach a final volume of 50 mL. A non-tissue sample was concurrently processed and utilized as a negative control. The samples from the mine tailings (exposed site) and soil (control site) were processed simultaneously in triplicate. All samples were cold stored (5 ± 2 °C) until HM quantification. Ultimately, metal levels were assessed through atomic absorption spectrophotometry utilizing the flame technique (GBC 908 A, GBC Scientific Equipment Pty Ltd., Victoria, Australia). The levels of Cd and Pb were measured through calibration curves created from standard solutions of pure metal ions (Agilent, Ultra Scientific, North Kingstown, RI, USA). The standard calibration curves documented correlation coefficients (R^2^) ranging from 0.995 to 1.0. The manufacturer’s specified minimum detection limits (mg L^−1^) for cadmium and lead are 0.0004 and 0.001, respectively. Average levels were indicated in mg∙kg^−1^.

### 4.9. Heavy Metal Translocation Index, Bioconcentration Factor, and Total Phytoextraction

The ability of *D. viscosa* for metal phytoextraction was assessed using two indexes: (1) the bioconcentration factor (BCF) was calculated using Equation (3), which assesses how effectively the plant accumulates metal from the substrate into its tissues [62], the bioavailable concentration of Cd and Pb in mine tailings of Huautla, Morelos was obtained from the study by Hernández-Flores [63]; and (2) the translocation factor (TF), which was calculated using equation number 4, assesses the efficacy of metal transport from the roots to the aerial portion of a plant [62]. If a plant presents FT values > 1, the species is considered an accumulator for the analyzed HM [62,64].

BCF = [HM]_leaf_/[HM]_mine tailings_ or [HM]_root_/[HM]_mine tailings_ (3)

FT = [HM]_leaf_/[HM]_root_ (4)

where:

[HM]_leaf_ = concentration of the HM detected in the leaf tissue
[HM]_mine tailing_ = bioavailable concentration HM in the mine tailings, and where
[HM]_root_ is the concentration of the heavy metal detected in the root tissue

Additionally, the phytoremediation ability of *D. viscosa* was assessed using a streamlined model suggested by Lam et al. [65] to categorize plant species as indicators, excluders, accumulators, and hyperaccumulators for soil remediation via phytoremediation. The researchers utilized characteristic curves derived from the concentrations of various metals (such as Cd and Pb) in soils and plants based on the experimental data (for more detail, see Lam et al. [65]). The simplified model uses an adjusted factor defined as [HM]_plant_/√[HM]_soil_ that must be compared with a threshold depending on the metal, type of measurement, and target (indicators, excluders, accumulators, and hyperaccumulators) [65].

Ultimately, the total phytoextraction potential (TPE) per hectare of *D. viscosa* for each heavy metal examined (Pb and Cd) after 12 months of treatment was calculated using Equation (5). For TPE determinations, the density of two populations of *D. viscosa* was estimated using a closed individual method described by Cottam and Curtis [66]. This study found a density of 10,806 individuals of *D. viscosa* per hectare.

TPE = ([HM]_root_ × BDW_root_) + ([HM]_leaf_ × BDW_leaf_] (5)

where:

TPE = the total phytoextraction for each HM [mg·ha^−1^]
[HM]_root_ = the HM concentration in root tissues [mg·kg^−1^ DW]
BDW_root_ = the dry weight of root biomass per hectare [kg·ha^−1^]
[HM]_leaf_ = the HM concentration in leaf tissues [mg·kg^−1^ DW]
BDW_leaf_ = the dry weight of leaf biomass per hectare [kg·ha^−1^]

### 4.10. Statistical Analysis

A two-way analysis of variance (ANOVA) was performed to evaluate the effect of the treatment (mine tailing and control), exposure time (2, 4, 6, 8, 10, and 12 months), and treatment × exposure time interaction on size, macro- and micro-morphological characters, physiological, and genetic damage in *D. viscosa*. Subsequently, a Tukey test was utilized to identify significant differences among pairs of average characters assessed across treatments [67]. Simple regressions were performed to determine the relationship between exposure time to the substrate and each analyzed character.

To evaluate the effect of treatment (control and exposed), exposure time (2, 4, 6, 8, 10, and 12 months), and treatment × time interaction on lead (Pb) and cadmium (Cd) concentration in root and leaf of *D. viscosa* a two-way analysis of variance was performed. Significant mean differences between treatments were determined with a Tukey multiple range test. Ultimately, simple regressions were executed to evaluate the relationship between exposure time to the substrate and the metal concentration in each tissue (root and leaf). STATISTICA 8.0 for Windows was used for all the statistical analyses [68].

## 5. Conclusions

This study shows that *D. viscosa* plants growing under in situ conditions on a substrate with HMs could bioaccumulate Cd and Pb in roots and leaves. These individuals showed high BCF values, which increased significantly over time. Moreover, BCF under in situ conditions were higher than those registered under greenhouse conditions. Therefore, *D. viscosa* could be considered a potential species for phytostabilization strategies of contaminated soils with Cd and Pb, which are very toxic. Moreover, this study suggests that *D. viscosa* could be proposed as a useful species in phytoremediation programs due to its ideal properties, such as (1) during its reproductive and vegetative stages, this species is not palatable, which reduces the risk of toxic HM biomagnification through the food chain, (2) wide geographic distribution, (3) potential to establish itself in HM contaminated soils, (4) high abundance, (5) Tolerant to HM mixtures in soils, (6) high BCF capacity for Cd and Pb. It is important to consider these parameters when selecting the plant species for phytoremediation of Cd and Pb-contaminated soils.

## Figures and Tables

**Table 1:** Multiple regression analysis testing the influence of Pb and Cd on macro- and micro-morphological characters, physiological, and genetic damage of *D. viscosa*. The number in bold indicates significant differences at *p* < 0.05 (in all cases, the degrees of freedom were 1).

Character	SS	*F*	*p*	% Variation
Height				
Cd	16,127.35	41.158	**0.000**	72.2
Pb	1637.61	4.179	**0.045**	7.3
Basal diameter				
Cd	44.78	5.449	**0.023**	9.6
Pb	149.66	18.212	**0.000**	31.9
Leaves number				
Cd	658.72	0.407	0.526	0.7
Pb	37,578.16	23.223	**0.000**	40.7
Dry root biomass				
Cd	354.35	50.882	**0.000**	79.2
Pb	97.21	13.959	**0.000**	14.5
Dry leaf biomass				
Cd	5692.76	31.205	**0.000**	54.7
Pb	644.73	3.534	0.065	6.2
Fresh root biomass				
Cd	1079.60	61.137	**0.000**	67.2
Pb	631.87	35.782	**0.000**	32.1
Fresh leaf biomass				
Cd	25,215.88	31.842	**0.000**	55.9
Pb	1932.44	2.440	0.123	4.2
Stomatal coverage				
Cd	2438.65	1.393	0.243	2.4
Pb	4497.32	2.568	0.114	4.5
Stomatal index				
Cd	35.20	0.315	0.577	0.5
Pb	0.25	0.002	0.962	0.0
Chlorophyll *a*				
Cd	32.14	1.478	0.229	2.6
Pb	0.00	0.000	0.988	0.0
Chlorophyll *b*				
Cd	15.25	1.84	0.179	3.2
Pb	8.66	1.047	0.310	1.8
Tail length				
Cd	2067.15	10.320	**0.002**	18.1
Pb	1065.73	5.453	**0.023**	9.3

**Table 2:** Average ± standard deviation of bioaccumulation, bioconcentration factor (BCF) and translocation factor values (FT) of Pb and Cd in root and leaves of *Dodonaea viscosa* from individuals growing under greenhouse conditions. *** = *p* < 0.001, n.s. = not significant differences.

	Concentration (mg∙kg^−1^)							
Metal	Time (Months)	N	Root	Leaf	BCF_root_	BCF_leaf_	TF	TF (Min–Max)
Cd								
	2	10	2.24 ± 1.15	1.47 ± 1.01	0.27 ± 0.14	0.18 ± 0.14	0.65 ± 0.62	(0.1–2.1)
	4	10	6.95 ± 2.93	3.89 ± 1.60	0.83 ± 0.35	0.46 ± 0.19	0.56 ± 0.66	(0.0–2.4)
	6	10	15.86 ± 3.01	5.17 ± 4.51	1.90 ± 0.36	0.62 ± 0.42	0.33 ± 0.31	(0.1–1.0)
	8	10	19.21 ± 6.28	10.07 ± 4.07	2.29 ± 0.75	1.20 ± 0.49	0.53 ± 0.17	(0.2–0.7)
	10	10	145.81 ± 18.75	76.97 ± 7.44	17.43 ± 2.24	9.20 ± 0.89	0.53 ± 0.10	(0.4–0.7)
	12	10	425.47 ± 24.67	192.57 ± 47.35	50.86 ± 6.56	23.02 ± 5.66	0.45 ± 0.11	(0.3–0.6)
Regression			*r* = 0.809, *r*^2^ = 0.649 ***	*r* = 0.798, *r*^2^ = 0.630 ***	*r* = 0.809, *r*^2^ = 0.649 ***	*r* = 0.798, *r*^2^ = 0.630 ***	*r* = −0.20, *r*^2^ = 0.025 n.s.	
Pb								
	2	10	4.37 ± 3.48	1.29 ± 0.98	0.63 ± 0.43	0.18 ± 0.09	0.29 ± 92.66	(0.1–293)
	4	10	35.67 ± 8.61	2.94 ± 1.63	5.12 ± 1.23	0.42 ± 0.23	0.08 ± 0.23	(0.0–0.8)
	6	10	929.50 ± 641.47	323.37 ± 370.35	133.3 ± 92.05	46.38 ± 33.12	0.35 ± 4.18	(0.4–13.5)
	8	10	1767.12 ± 1070.67	488.53 ± 249.04	253.48 ± 139.55	70.07 ± 43.10	0.28 ± 0.39	(0.1–1.0)
	10	10	4131.80 ± 382.71	1832.05 ± 115.29	592.67 ± 54.90	262.79 ± 16.54	0.44 ± 0.05	(0.4–0.5)
	12	10	6118.02 ± 1680.35	5568.63 ± 1032.19	877.58 ± 241.10	798.77 ± 148.06	0.99 ± 0.21	(0.7–1.5)
Regression			*r* = 0.875, *r*^2^ = 0.761 ***	*r* = 0.802, *r*^2^ = 0.636 ***	*r* = 0.875, *r*^2^ = 0.761 ***	*r =* 0.802, *r*^2^ = 0.636 ***	*r* = −0.19, *r*^2^ = 0.018 n.s.	

**Table 3:** Classification of *Dodonaea viscosa* into excluder, indicator, accumulator, and hyperaccumulator of Pb and Cd, using threshold values in mg∙kg^−1^. Hyp = Hyperaccumulator.

					Predicted for Pb and Cd				
Tissue	Metal	Sample []_soil_	Sample []_plant_	Adjusted AF	Excluder	Indicator	Accumulator	Hyperaccumulator	Results
Root	Pb	6.972	6118.02	2317.03					Hyp
	Cd	8.365	425.47	147.11	0.810	3.000	16.155	61.547	Hyp
Leaves									
	Pb	6.972	5568.63	2108.97	0.554	2.052	11.052	42.106	Hyp
	Cd	8.365	192.57	66.58					Hyp

**Table 4:** Macro- and micro-morphological characters, physiological, and genetic damage analyzed in *D. viscosa*.

Abbreviation	Character	Units
Macro-morphological		
TIH	Total individual height	cm
NL	Number of leaves	n
BD	Basal diameter	mm
DRB	Dry root biomass	g
FRB	Fresh root biomass	g
DLB	Dry leaf biomass	g
FLB	Fresh leaf biomass	g
Micro-morphological		
SI	Stomatal index	mm^2^
SC	Stomatal coverage	mm^2^
Physiological		
CaC	Chlorophyll *a* content	mg∙m^–2^
CbC	Chlorophyll *b* content	mg∙m^–2^
Genetic damage		
TL	Tail lenght	µ

## Data Availability

Please contact the author with a data request.
